# Gall Stones: Diagnosis by X-Rays

**Published:** 1914-06

**Authors:** 


**Gall Stones: Diagnosis by X-Rays.** Arial W. George and Isaac Gerber, Boston M. & S. Jour., April 30, 1914, state that, owing to the tendency to deposits of calcium salts even on cholesterin stones, the majority of biliary calculi, especially those causing trouble, are diagnosticable by this method.

